# Species-specific functions of Epstein-Barr virus nuclear antigen 2 (EBNA2) reveal dual roles for initiation and maintenance of B cell immortalization

**DOI:** 10.1371/journal.ppat.1006772

**Published:** 2017-12-20

**Authors:** Janine Mühe, Fred Wang

**Affiliations:** 1 Department of Medicine, Brigham & Women's Hospital, Boston, United States of America; 2 Department of Microbiology and Immunobiology, Harvard Medical School, Boston, United States of America; Baylor College of Medicine, UNITED STATES

## Abstract

Epstein-Barr virus (EBV) and related lymphocryptoviruses (LCV) from non-human primates infect B cells, transform their growth to facilitate life-long viral persistence in the host, and contribute to B cell oncogenesis. Co-evolution of LCV with their primate hosts has led to species-specificity so that LCVs preferentially immortalize B cells from their natural host *in vitro*. We investigated whether the master regulator of transcription, EBV nuclear antigen 2 (EBNA2), is involved in LCV species-specificity. Using recombinant EBVs, we show that EBNA2 orthologues of LCV isolated from chimpanzees, baboons, cynomolgus or rhesus macaques cannot replace EBV EBNA2 for the immortalization of human B cells. Thus, LCV species-specificity is functionally linked to viral proteins expressed during latent, growth-transforming infection. In addition, we identified three independent domains within EBNA2 that act through species-specific mechanisms. Importantly, the EBNA2 orthologues and species-specific EBNA2 domains separate unique roles for EBNA2 in the initiation of B cell immortalization from those responsible for maintaining the immortalized state. Investigating LCV species-specificity provides a novel approach to identify critical steps underlying EBV-induced B cell growth transformation, persistent infection, and oncogenesis.

## Author summary

Co-evolution of viruses and their natural hosts often results in barriers limiting the ability of viruses to infect hosts from other species. Understanding the nature of these species barriers has provided important insights into different aspects of viral life cycles, from virus entry into cells to host factors restricting viral replication. Epstein-Barr virus (EBV) infects nearly all adult humans and is associated with a variety of malignancies, including B cell lymphomas and epithelial cell cancers. Lymphocryptoviruses (LCV), i.e., EBV and its relatives, are also species-specific, but in this case we find LCV species-specificity to be associated with a different class of viral proteins, the latent infection proteins, that are associated with cell growth transformation and not a component of the virus particle or replication machinery. By investigating LCV species-specificity, we identified two distinct steps in the process of B cell immortalization which require EBNA2 activity. Furthermore, EBNA2 signals through one domain to initiate B cell immortalization and then utilizes a separate domain to maintain immortal B cell growth. Studying the species barrier for LCV B cell immortalization reveals important mechanisms into how EBV transforms B cell growth for life-long persistent infection and development of cancers.

## Introduction

Epstein-Barr virus is a gammaherpesvirus that naturally infects nearly all humans by adulthood, and once infected, the host harbors persistent EBV infection in a small fraction of B cells for life. Viral proteins expressed during latent infection of B cells are important for persistent EBV infection, contribute to malignant B cell proliferation and lymphoma development in humans, and immortalize B cells in tissue culture.

That EBV transforms B cell growth *in vitro* provides a valuable, tractable model system for dissecting the molecular mechanisms important for EBV’s ability to persist in humans and contribute to B cell malignancies [1]. The viral genes essential for EBV-induced B cell immortalization have been defined and their functions have been intensely investigated (reviewed in [2]). Thus, the overall strategy of EBV proteins manipulating host cell gene expression in favor of cell growth and survival is conceptually well established. For example, the Epstein-Barr virus nuclear antigen (EBNA) 2 interacts with various types of host cell proteins to regulate cellular and viral gene transcription. EBNA3A & -3C are additional viral nuclear proteins that act as transcriptional co-activators and repressors. The latent membrane protein (LMP) 1 is a constitutively active membrane receptor which acts as a potent activator of cell signaling pathways. Although much has been learned about these growth-transforming viral proteins, the reported repertoire of cellular pathways necessary for EBV-induced B cell immortalization is likely still incomplete, and little is known about the temporal requirements for activating these pathways during the process of EBV-induced B cell immortalization. Thus, new experimental approaches will be important for advancing our understanding of how EBV transforms B cells to the next level.

EBV-related gammaherpesviruses in the same lymphocryptovirus (LCV) genus naturally infect other hominoids (e.g., chimpanzees) and Old World non-human primates (OW-NHP, e.g., baboons and macaques), and their biology is virtually identical to EBV infection in humans. Notably, the natural host harbors persistent B cell infection for life, infection can be associated with B cell lymphomas, and LCVs immortalize B cells from their own natural host *in vitro* [3,4].

Hominoid and OW-NHP LCVs encode the same set of viral proteins as EBV, and their latent infection proteins appear to use the same molecular pathways as their EBV orthologues [5]. For example, LMP1 from baboon and rhesus LCV (rhLCV) interact with TRAFs through TRAF binding domains that are highly homologous to those in EBV LMP1 [6]. NHP-LCV EBNA3s interact with RBP-Jκ to act as transcriptional co-activators, and rhLCV EBNA2 (rhE2) transactivates the same viral promoters as EBV [7–9]. Similarly, the cellular pathways manipulated by LCV are highly conserved among OW-NHP and hominoids.

Despite the strong similarities among these viruses and their primate hosts, LCV-induced B cell immortalization is species-specific. OW-NHP LCV cannot immortalize B cells derived from hominoids, and EBV cannot immortalize B cells from OW-NHP [10,11]. We have previously shown that virus entry is not a barrier for cross-species infection, since OW-NHP LCV can enter human B cells [11]. In contrast to the well conserved viral entry proteins, the latent infection proteins associated with growth transformation are the most dynamically evolving genes in LCVs, suggesting ongoing adaptation of these viral proteins to evolutionary changes in the primate host [5]. We hypothesized that LCV latent infection proteins evolved to manipulate molecular pathways essential for B cell immortalization in the natural host, but may have lost the ability to manipulate the same pathways in other primate species, e.g., rhLCV latent infection proteins evolved to specifically interact with rhesus macaque host proteins and fail to immortalize human B cells because they cannot properly interact with the related human proteins. Thus, species-specificity is intrinsically linked to functions required for EBV-induced B cell immortalization and can reveal novel cell pathways manipulated by EBV latent proteins.

## Results

### rEBVs expressing EBNA2 proteins from nonhuman primate LCVs

**Fig 1:**
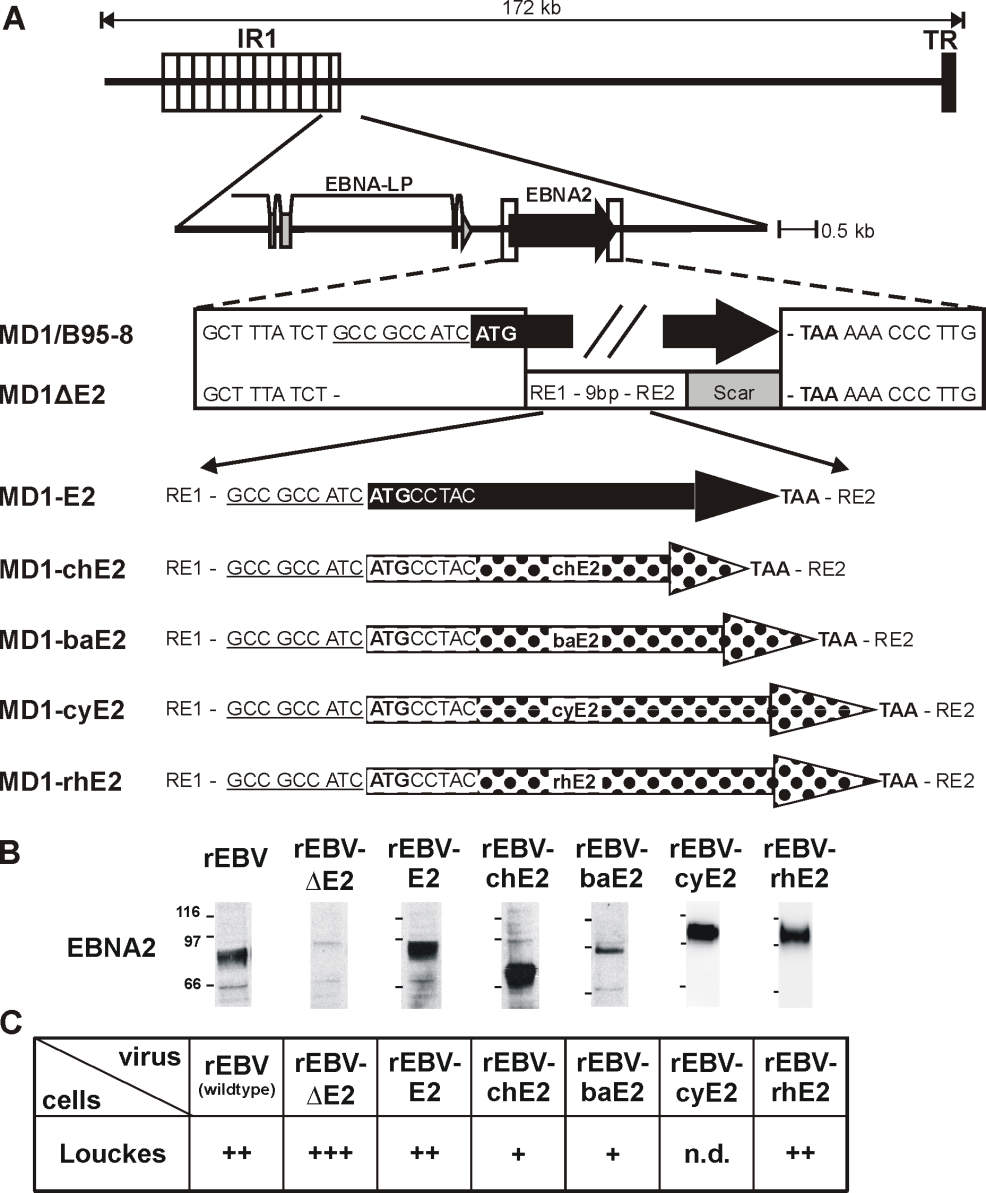
Recombinant EBVs expressing NHP EBNA2 proteins infect human B cells. A) Relative location of the EBNA2 gene locus downstream of the major Internal Repeat (IR1) within the EBV genome and detailed description of the flanking regions within the EBV BAC (MD1). The native flanking sequences and EBNA2 open reading frame (black arrow) are shown in the wild type EBV BAC derived from B95-8 (MD1/B95-5). The EBNA2 deleted BAC (MD1ΔE2) contains the linker region from the shuttle plasmid containing two restriction enzyme sites (RE1 and 2) and a downstream scar sequence as a result of the cloning procedure. Recombinant EBVs carrying EBV E2 (MD1-E2, black arrow), chimpanzee, baboon, cynomolgus or rhesus LCV EBNA2 (MD1-chE2, -baE2, -cyE2, -rhE2; polka dots) were cloned by recombining similar DNA fragments from shuttle plasmids containing each EBNA2 species. In this manner all rEBV contained an EBNA2 species with identical upstream and downstream sequences. B) Production of rEBVs was confirmed by infection of EBV-negative Louckes cells and detection of EBNA2. The monoclonal antibody against EBNA2 (PE2) was used to detect LCV EBNA2 expression by immunoprecipitation and subsequent immunoblotting from cell lysates prepared 2 days after infection with equal volumes of rEBV. C) Relative infectious titers of rEBV supernatants were determined by infection of EBV-negative Louckes cells and puromycin selection. Louckes cells were infected with equal volumes of rEBV, distributed into 50 microtiter wells, and selected for rEBV infection using puromycin. After 3–4 weeks the number of wells with cell growth was counted, and average results for at least 2 independent supernatants are shown. +: 1–20 wells with growth/50 total wells, ++: 21-44/50, +++: 45-50/50, n.d.: not done.

We asked whether NHP LCV EBNA2s are capable of functionally replacing EBV EBNA2 (E2) for human B cell immortalization by constructing recombinant Epstein-Barr viruses (rEBVs) carrying chimpanzee- (chE2), baboon- (baE2), cynomolgus- (cyE2), or rhesus LCV EBNA2 (rhE2) (Fig 1A). A rEBV with the EBNA2 open reading frame (ORF) deleted (rEBVΔE2) was used as a negative control. Successful production of recombinant viruses (rEBVs) was confirmed by infection of EBV-negative human B lymphoma cells (Louckes). All EBNA2s could be detected in Louckes with the expected relative molecular weight for the respective NHP LCV species 2 days post infection (Fig 1B). We used the monoclonal EBNA2-specific antibody PE2, since it cross-reacts with NHP EBNA2s and detects comparable levels in LCL derived by NHP LCV infection of B cells from their natural hosts [11]. Infection of Louckes cells with viral supernatants followed by selection with puromycin confirmed presence of infectious rEBV, and the relative amounts of infectious virus detected is shown (Fig 1C).

### rEBV infection of ER/EB2-5 cells: Maintenance of B cell immortalization

NHP EBNA2s were tested for their ability to maintain growth of EBV-immortalized B cells by superinfection of ER/EB2-5 cells with rEBVs. ER/EB2-5 cells are human B cells conditionally immortalized by co-infection of the EBNA2-deletion virus P3HR-1 EBV with a mini-EBV carrying an EBNA2 fused to an estrogen-receptor (ER-EBNA2) [12]. In these cells, EBNA2 function and continued B cell growth is dependent on estrogen, and withdrawal of estrogen from the culture medium leads to loss of EBNA2 function and cell death. Providing an estrogen-independent EBNA2 in *trans* can rescue cell growth in estrogen free media, but only if the EBNA2 protein is functional for maintaining human B cell immortalization [13].

**Fig 2:**
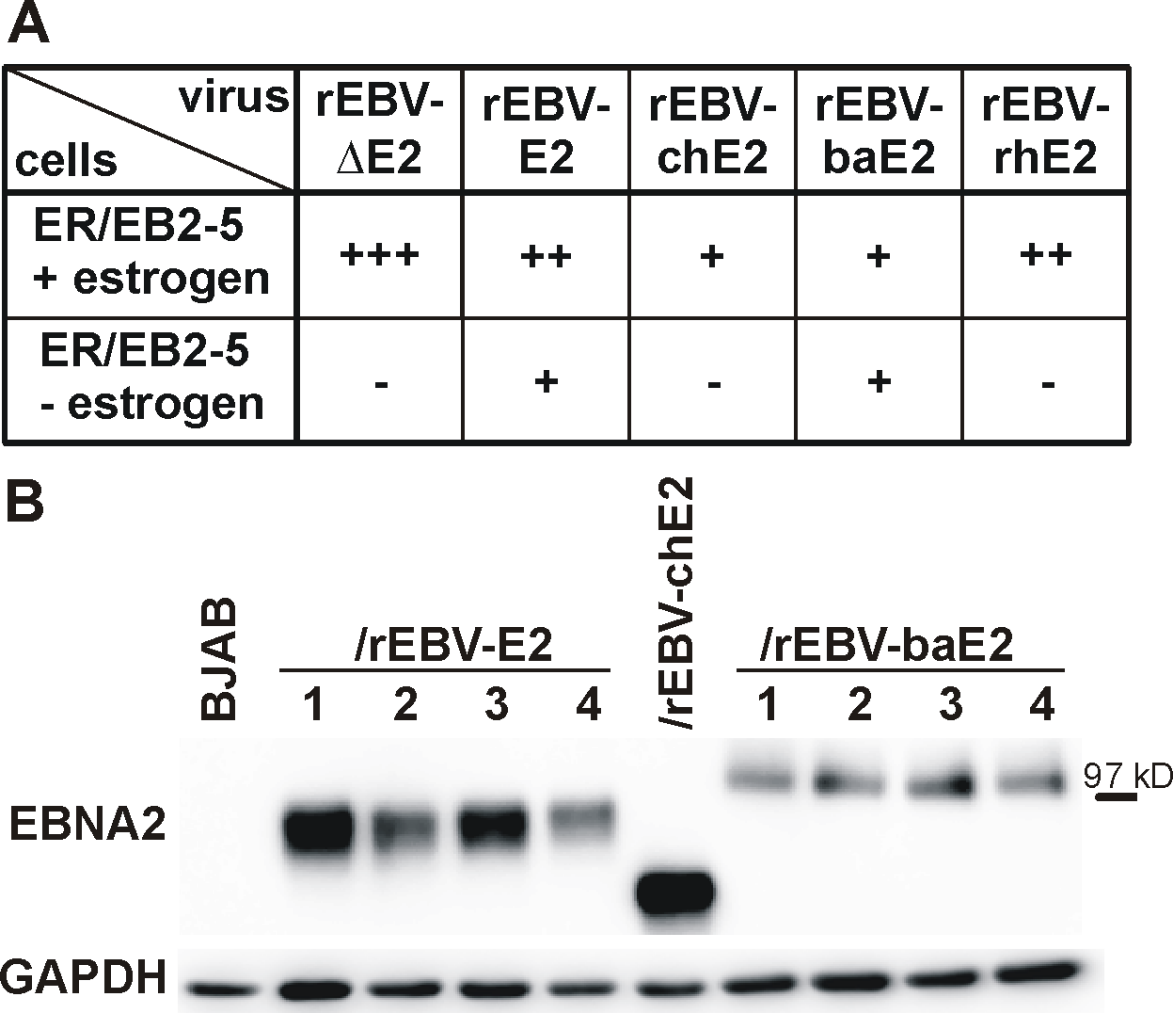
Maintenance of B cell immortalization in conditionally immortalized ER/EB2-5 cells by rEBVs. A) ER/EB2-5 cells were infected with rEBVs and distributed into 100 microtiter wells. Half the wells were selected for infection by culture with medium containing puromycin and estrogen (+estrogen) and the other half of the cells underwent biological selection in medium without additives (-estrogen) to determine functionality of the EBNA2 protein. After 6 weeks the number of wells with cell growth was determined. The average result from multiple experiments is shown with 2 independent viral supernatants of each rEBV tested. +: 1–20 wells with growth/50 total wells, ++: 21-44/50, +++: 45-50/50, -: no growth. B) Four weeks after infection, selected clones growing in the absence of estrogen were used to detect EBNA2 and GAPDH as loading control by immunoblot. Cells infected with rEBV-E2, rEBV-chE2, or rEBV-baE2 express an EBNA2 species of the relative molecular weight expected for the respective rEBV.

Successful superinfection of ER/EB2-5 cells with each rEBV was confirmed by selection for puromycin resistance in the presence of estrogen (Fig 2A, top row). The relative amounts of infectious rEBVs detected in ER/EB2-5 cells were comparable to those detected after rEBV infection of Louckes cells (Fig 1C).

To functionally test whether NHP EBNA2s could maintain B cell immortalization, parallel infections of ER/EB2-5 cells with rEBVs were cultured without estrogen or puromycin (Fig 2A, bottom row). As expected, cell growth was not detected in any wells containing ER/EB2-5 cells superinfected with rEBVΔE2, whereas rEBV-E2 superinfection readily rescued ER/EB2-5 cell growth, e.g., 16 of 50 positive microtiter wells in one experiment (average results from multiple experiments shown in Fig 2A). Superinfection with rEBV-baE2 resulted in 14 of 50 wells with cell growth after 6 weeks in culture without estrogen in the same experiment. Immunoblot analyses of immortalized cells expanded from independent wells confirmed expression of either EBV E2 or baE2 from the superinfecting rEBV (Fig 2B). These results indicate that baE2 can functionally replace EBV E2 for the maintenance of EBV-dependent B cell immortalization, and the similar number of rescued wells suggests that the activity of baE2 and EBV E2 may be comparable in ER/EB2-5 cells which could be confirmed with more quantitative experiments.

Recombinant EBV-chE2 was also able to rescue growth in a single well at 4 weeks, and expression of chE2 was confirmed by immunoblot analyses of cells expanded without estrogen (Fig 2B). However, ER/EB2-5 cells superinfected with rEBV-chE2 could not be expanded and sustained for prolonged periods in culture without estrogen. In contrast, rEBV-E2 and rEBV-baE2 superinfected cells could be readily expanded and sustained in culture without estrogen for at least 4 months. The weak chE2 phenotype cannot be fully explained by low amounts of virus since there were comparable numbers of ER/EB2-5 wells detected after infection with rEBV-baE2 and rEBV-chE2 in the presence of estrogen and puromycin. In contrast to rEBV-chE2 and rEBV-baE2, there was no evidence for rescue of ER/EB2-5 cell growth by rEBV-rhE2 superinfection despite readily detectable amounts of infectious virus. These results indicate that rhE2 functions in a species-specific manner, i.e., it can immortalize rhesus B cells, but it cannot maintain immortalization of human B cells. In contrast, baE2, and to some extent chE2, can function in human B cells to maintain EBV immortalization.

### rEBV infection of primary B cells: Initiation and maintenance of B cell immortalization

**Fig 3:**
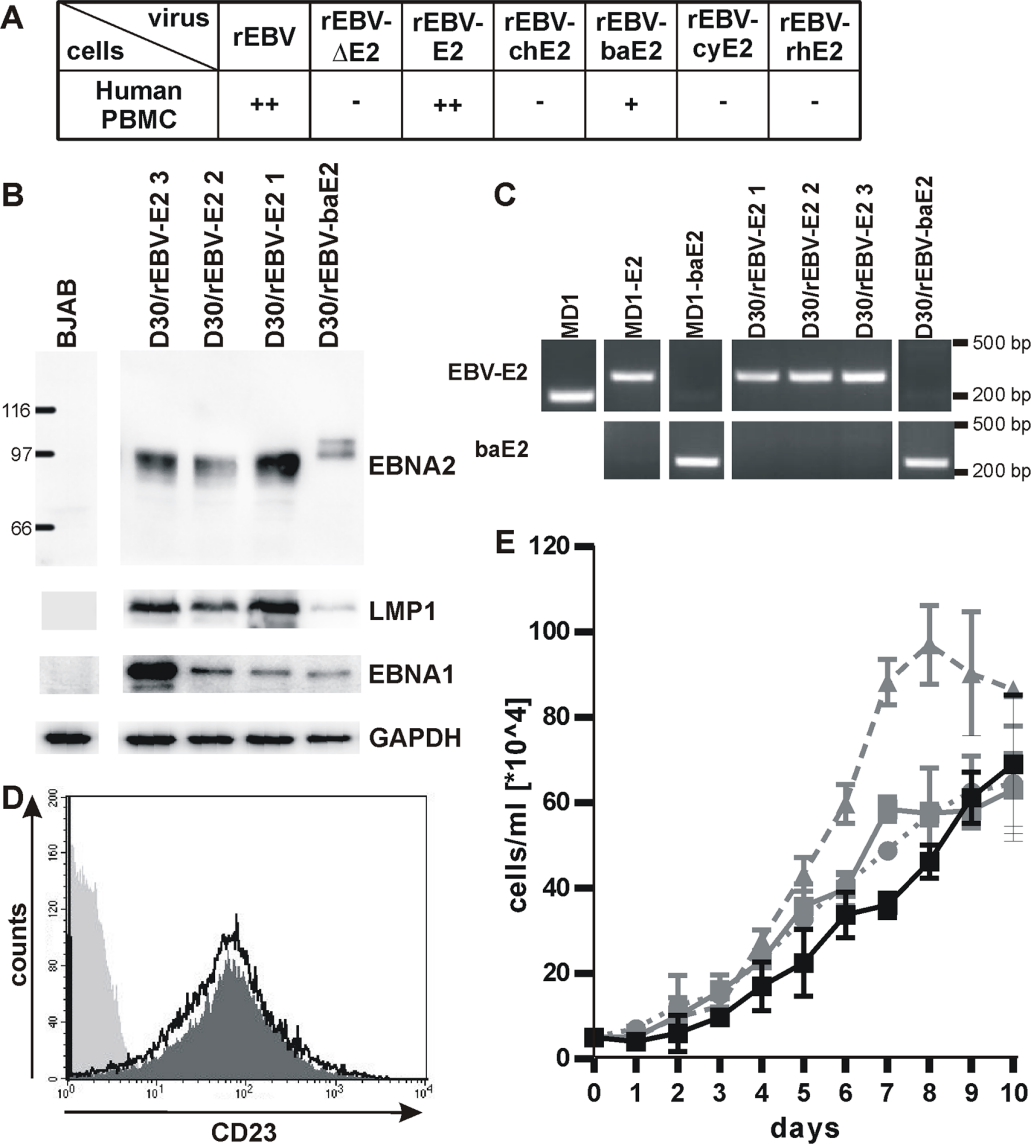
*De novo* immortalization of human PBMC by rEBVs. A) Human PBMC were infected with rEBVs, cultured in 50 microtiter wells, and outgrowth of immortalized cells was quantified 6 weeks after infection. Average results for at least 2 experiments are shown using independent virus preparations of rEBV (n = 3), rEBVΔE2 (n = 3), rEBV-E2 (n = 5), rEBV-chE2 (n = 4), rEBV-baE2 (n = 3), rEBV-cyE2 (n = 2), and rEBV-rhE2 (n = 4). +: 1–20 wells with growth/50 total wells, ++: 21-44/50, -: no growth. B) Representative cell lines derived from PBMC of the same donor (D30) infected with rEBV-E2 and rEBV-baE2 were immunoblotted for detection of EBNA2, EBV LMP1, and EBV EBNA1. Detection of GAPDH controlled for loading and BJAB cells served as EBV-negative control. C) Expression of only one EBNA2 species was confirmed by species-specific PCR amplification for EBV or baboon LCV EBNA2 in rEBV immortalized cells. Wild type MD1, MD1-E2, and MD1-baE2 BAC DNAs purified from bacteria served as controls. D) Surface expression of the EBNA2-regulated marker CD23 on rEBV immortalized cells D30/rEBV-baE2 (dark grey fill), and D30/rEBV-E2 1 (black line) or the EBV negative cell line BJAB (light grey fill) was determined by flow cytometry. E) Growth curve of D30 LCLs transformed with rEBV-E2 (grey curves, line: clone 1, dashes: 2, dots: 3) or rEBV-baE2 (black line).

Peripheral blood mononuclear cells (PBMC) were infected with rEBVs to test NHP EBNA2s for their ability to induce B cell immortalization *de novo*. Wild type rEBV and rEBV-E2 virus stocks efficiently immortalized human PBMC with 32–50 and 25–48 of 50 positive microtiter wells in multiple experiments (Fig 3A), and as expected, rEBVΔE2 was incapable of immortalizing human PBMC. No growth was observed after infection of human PBMC with rEBV-chE2, -cyE2, or -rhE2 virus stocks. However, a single positive well was obtained by infection with rEBV-baE2, and immunoblot detection of baE2 concomitant with EBV LMP1 and EBNA1 expression indicated the LCL was immortalized by rEBV-baE2 (Fig 3B). PCR analysis showed no evidence of spurious co-infection with wild type EBV, and no EBV E2 DNA could be detected in cells immortalized with rEBV-baE2 (Fig 3C). Recombinant EBV-baE2 cells expressed similar cell surface levels of the B cell activation antigen, CD23 (Fig 3D), and grew at comparable rates as rEBV-E2 infected cells (Fig 3E). The *de novo* immortalizing activity of rEBV-baE2 could be confirmed in 2 additional infections of human PBMC from different donors (with 2/50 and 1/50 positive/total infected wells).

Thus, rEBVs showed that all NHP EBNA2s are impaired for immortalization of primary human B cells and thus, are species-specific in their ability for *de novo* immortalization. Recombinant EBV-baE2 was different from the others since a minimal level of *de novo* immortalization could be detected, far below that of wild type EBV E2. This scenario suggests that *de novo* immortalization may consist of two steps, i.e., an initiation step followed by maintenance. While rEBV-baE2 readily maintained B cell immortalization in the ER/EB2-5 cell assay, the weak *de novo* immortalization of primary B cells suggest impaired initiation as the rate limiting, i.e., species-specific, step. It is impossible to determine from these studies whether ch-, rh-, and cyE2 are also impaired for initiation since they have a complete, or near-complete, inability to maintain B cell immortalization. The unique species-specific phenotype of baE2 provides evidence for different functional roles of EBNA2 during two distinct phases of B cell immortalization, i.e., initiation and maintenance.

### Relative transactivating activity of NHP EBNA2s in human B cells

**Fig 4:**
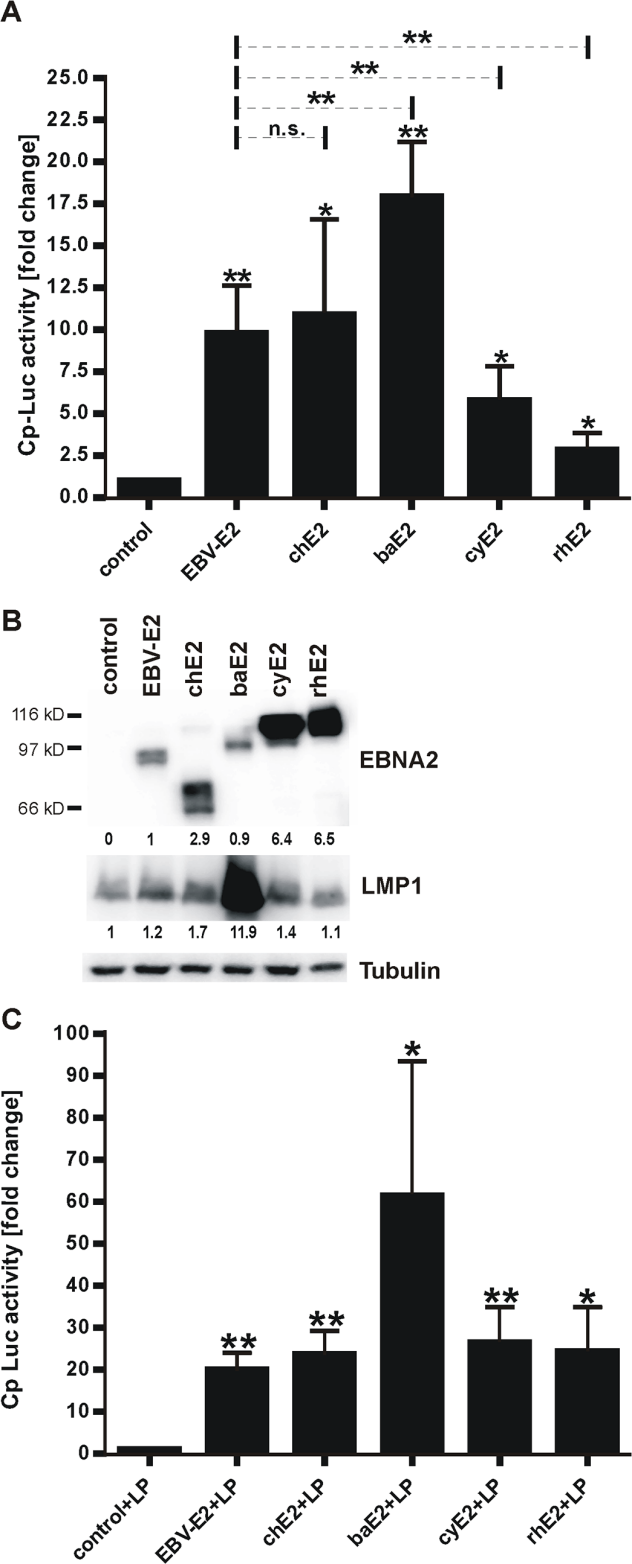
Transactivation activity of NHP EBNA2s in human B cells. P3HR-1 cells were transfected with a Cp-Luciferase reporter, a Renilla control, and EBNA2 expression plasmids or empty control. A) 48h after transfection Luciferase and Renilla levels were measured and normalized to Renilla. Cp transactivation levels shown represent fold change over control transfection. Asterisks above standard deviation bars indicate significance relative to control, and asterisks above the dotted lines indicate significance relative to EBV E2. * p<0.05, ** p<0.01, n.s. not significant, n = 4. B) EBNA2 and LMP1 were quantified from a representative reporter assay by immunoblot, and tubulin was used as loading control. Relative expression levels of EBNA2 normalized to EBV E2 sample and LMP1 normalized to levels of control transfection are quantified under each blot. C) Cp activity in response to EBNA2 and EBNA-LP co-expression was analyzed as described for panel A) with co-transfection of an EBNA-LP expression plasmid. Luciferase levels represent fold change over LP alone (control+LP).

We investigated whether there were quantitative differences in the ability of NHP EBNA2s to transactivate viral promoters in human cells that might reflect species-specific functions. Co-transfection of EBV-, ch-, ba-, cy-, or rhE2 expression plasmids with a Cp driven Luciferase reporter all induced Luciferase expression significantly over background control confirming transactivation activity of all NHP E2s in human B cells (Fig 4A). However, there were considerable differences in the relative amounts of transcriptional activation by NHP EBNA2s. Cy- and rhE2 were significantly less active than EBV E2 and only induced Luciferase responses 5.8 and 2.9 fold over background compared to 9.8 fold for EBV E2 (average response of 3 independent experiments, p<0.01). ChE2 activity was not different from that of EBV E2 (10.9 fold), and baE2 was notably better than all other EBNA2 species (17.9 fold; p<0.01).

Immunoblots showed cy- and rhE2 were expressed at levels much higher than EBV E2 (5.7–8.6 fold higher than EBV E2 levels; Fig 4B), suggesting an even greater functional deficit compared to EBV E2 when corrected for expression levels. ChE2 was frequently expressed at levels slightly greater than EBV E2, raising the possibility that chE2 may not be fully comparable to EBV E2 when corrected for expression levels. BaE2 expression was often equal to or below that of EBV E2 (0.3–4 fold difference to EBV E2 levels) with consistently higher activity supporting the interpretation that baE2 is equal to or more active than EBV E2.

Interestingly, baE2 also strongly induced LMP1 expression in P3HR-1 cells better than any other EBNA2 species including EBV E2 (Fig 4B). BaE2 induced a 4–12 fold induction of LMP1 levels over control transfection (mean = 8.6). EBV E2 and chE2 induced comparable LMP1 expression (1.3 and 1.9 fold), and cyE2 and rhE2 were impaired with only 1.2 fold average LMP1 induction for cyE2 and no changes in LMP1 expression with rhE2. Thus, the relative transactivating activity of NHP EBNA2s in human B cells correlated with their relative ability to maintain growth of ER/EB2-5 cells, i.e., baE2 was the most active, cy- and rhE2 were the least active, and chE2 showed intermediate activity.

When EBNA-LP was co-transfected with EBNA2 into P3HR-1 cells, Cp activity was markedly increased versus EBNA2 alone in each case, indicating that all EBNA2 species were capable of interacting and co-activating with EBV EBNA-LP (Fig 4C). EBNA-LP alone induced luciferase expression by 6.8 fold (average of 4 experiments). Addition of EBV E2 or chE2 increased luciferase levels by 20 and 24 fold. Co-expression of cy- and rhE2 with EBNA-LP resulted in Cp activity levels comparable to that of EBV E2 (26 and 24 fold). BaE2 was still superior to all other EBNA2s with an additive effect of 61 fold induction. EBV EBNA-LP has previously been reported to enhance rhE2 transactivation, and our findings with ch-, ba-, and cyE2 provide additional evidence that the interaction of NHP EBNA2s with EBV EBNA-LP is not species-specific [9].

### EBNA2 chimeras reveal domains associated with species-specific functions: Cp transactivation

**Fig 5:**
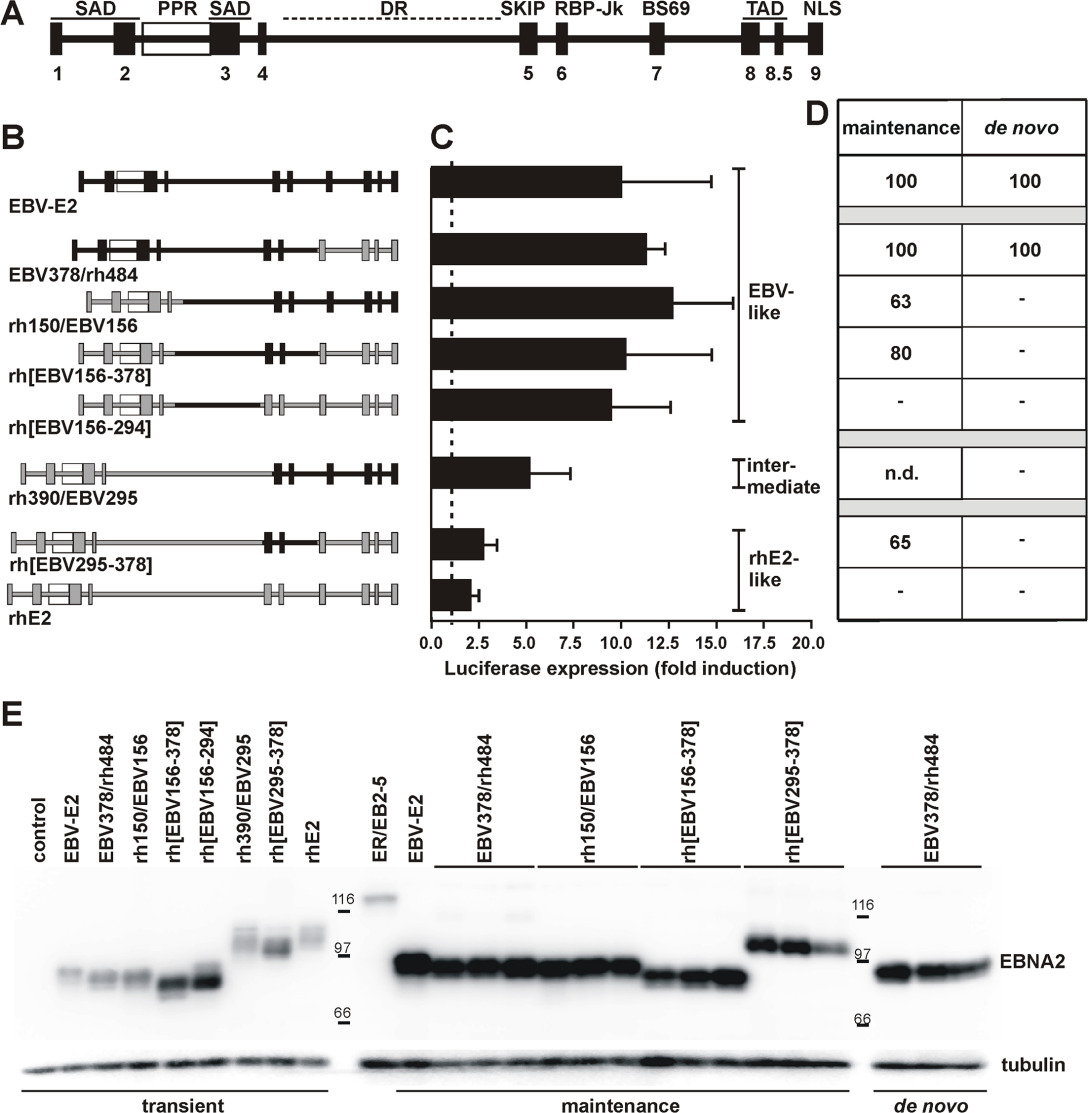
Activity of EBV/rhLCV-EBNA2 chimeras in Cp transactivation, maintenance, and *de novo* immortalization. A) Schematic representation of EBV E2 with its 10 Conserved Regions (CRs, black boxes), the poly-proline region (PPR), and diversity region (DR). Functional domains include self association domains (SAD), binding sites for SKIP, RBP-Jκ and BS69, transactivation domain (TAD) and a nuclear localization signal (NLS). B) Overview of EBNA2 chimeras used in this study showing portions derived from EBV E2 in black and those from rhE2 in grey. Relative sizes of chimeras are drawn to scale. Chimeras are labeled with the last or the first amino acid of the fragments that were fused together corresponding to the position within the wild type EBV E2 or rhE2, e.g., EBV378/rh484 is a fusion of the EBV E2 fragment 1–378 and the rhE2 fragment 484–605. For reference, position 378/379 of EBV E2 aligns with position 483/484 in rhE2. Chimeras labeled with brackets indicate internal exchanges, e.g., rh[EBV156-378] is a rhE2 mutant that expresses the EBV E2 region 156–378 in place of the corresponding rhE2 sequences. C) Transactivation activity of E2 chimeras was determined in P3HR-1 cells after transfection with expression plasmids for E2 chimeras and a Cp-Luciferase reporter plasmid. Two days after transfection Luciferase levels were measured and normalized to levels of internal Renilla controls. Activity is expressed as fold change over transfection with empty control plasmid (dashed line). All chimeras induced Cp activity significantly above background (p<0.05, n≥3). D) Recombinant EBVs expressing E2 chimeras were used to infect ER/EB2-5 cells or human PBMC as in Figs 2 and 3 to test their ability to rescue ER/EB2-5 cell growth (maintenance) or immortalize human PBMC (*de novo*). After infection ER/EB2-5 cells were plated into 2 microtiter plates (50 wells each) and selected with puromycin in the presence of estrogen, or cultured in medium without additives. After 6 weeks the number of wells with cell growth was counted and percent outgrowth determined (average of 2 infections using independent virus preparations). For maintenance results are relative to control infection (number of wells without estrogen/number of wells with estrogen with ‘-‘ indicating no growth without estrogen, but growth with estrogen). E) EBNA2 expression was confirmed by immunoblot with PE2 antibody from 2x10^5^ transiently transfected P3HR-1 cells described in panel C) or selected ER/EB2-5 or LCL clones infected with rEBVs described in panel D). Uninfected ER/EB2-5 cells grown with estrogen served as a control for ER-EBNA2 expression.

Since rhE2 demonstrated the strongest species-specific effects in maintenance, *de novo* immortalization, and transcriptional activation, we constructed chimeras to determine which regions from EBV E2 could restore activity to rhE2 in human B cells. EBV- and rhE2 share only 47% amino acid similarity, but overall they share common structural features (Fig 5A). Both proteins have a large central Diversity Region (DR) that is defined by sequence divergence between EBNA2 alleles from different LCV strains and species, i.e., type 1 and 2 of EBV or rhLCV [14]. Alignment of LCV EBNA2 sequences has also revealed 10 Conserved Regions (CR) with nearly identical amino acid sequences (Fig 5A) [11]. Various EBNA2 functions have been linked to these conserved sequence domains, e.g., self association to CR1-3, interaction with transcription factors such as SKIP and RBP-Jκ to CR5 and CR6, recruitment of the general transcription machinery including TFIIB, TFIIH, and TAF40 to the acidic residues in CR8 and CR8.5, and nuclear localization to CR9 (Fig 5A) [15–21]. We divided EBNA2 into 4 parts based on these common features between EBV and rhLCV E2: i) the N-terminus containing CR1-4, ii) DR, iii) CR5-6, and iv) the C-terminus with CR7-9. Chimeric EBNA2 proteins were constructed composed of parts from EBV- and rhE2 (Fig 5B).

All chimeras localized exclusively to the nucleus (S1 Fig), were expressed at comparable levels (Fig 5E) and were able to transactivate Cp significantly above background in human cells indicating that chimeric EBNA2 proteins were expressed with appropriate conformational integrity for functional activity (Fig 5C). Four chimeras induced Cp activity to EBV E2 levels, one chimera induced Cp at low levels comparable to rhE2, and one chimera induced Cp at intermediate levels in human B cells. The 4 chimeras that induced EBV E2-like Cp levels all contained the EBV DR and replacing the rhE2 DR with the EBV DR (rh[EBV156-294]) was sufficient to confer EBV-like Cp activity to rhE2. These results indicate that the EBV DR is sufficient for full transactivation of Cp in human cells and contributes to this transactivation activity in a species-specific manner.

**Fig 6:**
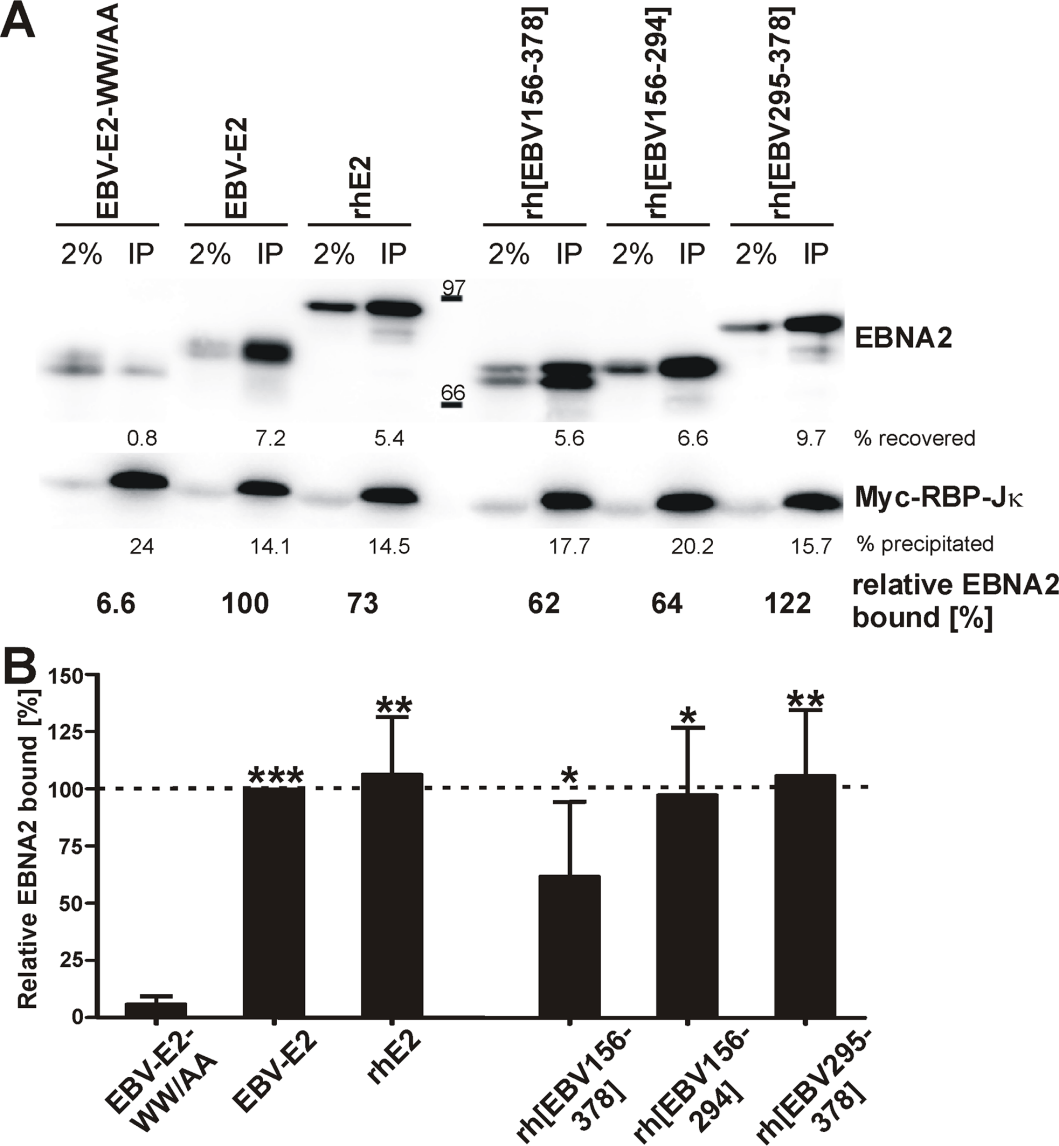
Interaction of EBNA2s with human RBP-Jκ. 293T cells were transfected with a myc-tagged RBP-Jκ expression plasmid. After 72h cells were lysed, and combined with comparable amounts of *in vitro* transcribed and translated EBNA2 proteins. The RBP-Jκ/EBNA2 complexes were isolated by co-immunoprecipitation with an anti-myc-antibody. EBNA2 and myc-RBP-Jκ were detected and quantified by immunoblot. A) Immunoblot results of a representative experiment showing EBNA2 and myc-RBP-Jκ detection from 2% of input (left lane) and 100% of precipitates (IP, right lane). EBV-E2-WW/AA is a RBP-Jκ binding mutant. Quantification of recovered EBNA2, precipitated myc-RBP-Jκ, and relative amount of bound EBNA2 in this experiment is shown below immunoblots. B) Average results for 4 independent experiments are shown with the amount of EBV E2 bound to RBP-Jκ set to 100%; for rhE2[EBV156-294] n = 3. Asterisks indicate significance relative to EBV-E2-WW/AA background. * p<0.05, ** p<0.01, *** p<0.001.

Since binding to RBP-Jκ is crucial for recruitment of EBNA2 and Cp transactivation, we asked whether rhE2 can bind to human RBP-Jκ and whether EBV DR in the rhE2 chimera alters the RBP-Jκ interaction [22]. Wild type EBV E2, rhE2, and EBNA2 chimeras containing EBV DR with CR5-6 (rh[EBV156-378]), DR alone (rh[EBV156-294]), or CR5-6 alone (rh[EBV295-378]) were produced by coupled *in vitro* transcription/translation and mixed with lysates of myc-RBP-Jκ expressing 293T cells. Myc-RBP-Jκ was immunoprecipitated, and the amount of bound EBNA2 was detected by immunoblot (Fig 6A) and quantitated (Fig 6B). An EBV E2 mutant with a RBP-Jκ binding site mutation (WW323/324AA) was not co-precipitated with myc-RBP-Jκ (average of 6% residual binding) and served as background control. All EBNA2s tested bound RBP-Jκ significantly better than the control. In addition, rhE2 and EBNA2 chimeras showed a 97–106% binding efficiency when compared to EBV E2 (100%, Fig 6B). Rh[EBV156-378] was slightly decreased in its ability to interact with RBP-Jκ (average of 62%), but this difference from EBV E2 was not significant. Thus, the interaction of EBNA2s with human RBP-Jκ is not species-specific, and the restoration of high level Cp transactivation by EBV DR in rhE2 chimeras is not associated with a change in RBP-Jκ interaction.

### EBNA2 chimeras: Initiation and maintenance of immortalization

Next, chimeras were introduced into rEBVs and tested for their ability to maintain ER/EB2-5 cell growth. Comparable amounts of infectious rEBVs containing chimeric EBNA2s were readily detected after infection of ER/EB2-5 cells, since outgrowth could be observed in 60–100% of wells in the presence of estrogen and puromycin. In the absence of estrogen, all E2 chimeras tested were able to maintain ER/EB2-5 growth (Fig 5D), except for rh[EBV156-294] which could not rescue ER/EB2-5 cells, even though the inclusion of EBV DR was sufficient to support EBV E2-like transactivation. Thus, EBV E2-like transactivation was not sufficient to maintain B cell immortalization. All other chimeras contained the EBV CR5-6 region, and rhE2 CR5-6 was present in the only E2 proteins (wild type rhE2 and rh[EBV156-294]) unable to maintain immortalization. Expression of E2 chimeras was detected by immunoblot from ER/EB2-5 cell clones maintained by recombinant EBV. All clones carrying one of the four different chimeric E2s containing EBV CR5-6 (Fig 5E) expressed a protein of the expected size. This suggests that the CR5-6 region functions in a species-specific manner to help maintain B cell immortalization.

When rEBVs expressing E2 chimeras were used to infect human PBMCs, only one chimera was able to support immortalization of primary human B cells (EBV378/rh484, Fig 5D), indicating that the C-terminal part of rhE2 containing CR7-9 was fully functional for *de novo* human B cell immortalization and did not function in a species-specific manner. The other 3 chimeras capable of maintaining ER/EB2-5 growth were all unable to immortalize human PBMCs, and the only region in common among these three chimeras was the rhE2 amino terminus, i.e., the rhE2 CR1-4 region. In the case of rh150/EBV156, the chimeric EBNA2 contained only the 150 amino terminal residues from rhE2 and was unable to immortalize human PBMC *de novo*. This negative result was not due to a nonspecific adverse effect of fusing the amino terminus of rhE2 to EBV E2 since the same recombinant EBV was capable of maintaining growth of ER/EB2-5 cells and the chimeric EBNA2 was capable of inducing EBV E2-like transactivation. This rhE2 chimera reproduces the maintenance competent/initiation defective phenotype of baE2 and maps a species-specific effect for initiation to the amino terminal region of EBNA2.

## Discussion

In these studies, we showed that the EBNA2 proteins from closely related lymphocryptoviruses are species-specific for B cell immortalization. Furthermore, by investigating the species-specific effects of NHP LCV EBNA2s in human B cells, we were able to discover that EBNA2 contributes to B cell immortalization through two distinct mechanistic steps that we refer to as initiation and maintenance. EBNA2 likely manipulates many different cell pathways to successfully induce and maintain B cell immortalization, some of which may be common to both steps and some of which may be unique. We were able to reveal these two steps in B cell immortalization by investigating the species-specificity of closely related LCV EBNA2s, e.g., baE2, and by mapping species-specific functions for each step to different EBNA2 domains.

Species-specificity is a result of virus adaptation to its natural host, and genetic differences between hosts can create barriers to cross-species infection at any point of the viral life cycle. Species-specific barriers to viral entry are common, e.g., preference of human or avian Influenza viruses for different sialic acid species and Hepatitis C virus restriction to humans and chimpanzees [23,24]. Similarly, interactions with host cell proteins important for virus replication can create a species-specific barrier, e.g., requirement of the human DNA repair protein Nbs1 for Herpes Simplex virus 1 replication [25]. Species-specific barriers can also be created by cellular host restriction factors such as Trim5α, or protein kinase R (PKR) which detect and inhibit viral components [26]. Viral strategies to avoid such factors include constant evolution of the capsid protein by Simian Immunodeficiency viruses to prevent Trim5α binding or expression of a PKR inhibitor, like human Cytomegalovirus’ TRS1 protein [27,28].

In contrast, the barrier to LCV cross-species infection is associated with latent infection, and not virus binding, entry, or replication. The current studies prove that at least one level of the LCV species-specificity affects the latent infection protein EBNA2. The latent infection proteins show the greatest sequence divergence between LCV species indicating ongoing evolution and adaptation to the respective hosts. However, most of the cell proteins known to interact with EBV latent proteins are fundamental growth associated proteins that are highly conserved among primate species making them unlikely candidates for driving this degree of virus evolution. Since LCVs are co-evolving with their primate hosts to preserve the ability to immortalize B cells, the biological drivers of LCV species-specificity are by default associated with pathways essential for the virus. Thus, understanding the underlying mechanisms of EBNA2 species-specificity is likely to reveal novel pathways critical for EBNA2 function.

Recent studies focusing on the early events after EBV infection of resting B cells have described dramatic virus-induced changes not evident in immortalized cell lines, e.g., rapid increase in cell size and an early hyperproliferative state [29]. Our studies identify a functional role for an EBNA2 dependent pathway essential for the early stages of EBV infection and the initiation of B cell immortalization that is independent of its functions later on. It is intuitive that EBV may be required to overcome different barriers in *de novo* immortalization of a resting B cell versus maintenance of a (conditionally) immortalized cell. In a resting B cell, the virus must first reprogram the cell from a quiescent state into an active, proliferating state. In the fully immortalized B cell, the host genome may already be epigenetically programmed for proliferation, and viral proteins may only need to sustain transactivation of a subset of host cell genes required for maintenance. During the early phases of EBV infection in resting B cells, EBNA2 likely contributes to the reprogramming of the resting state into a proliferative state. The interaction of EBNA2 with chromatin remodelers and EBNA2’s ability to act as a pioneering co-factor for transcription factors such as EBF1 and RBP-Jκ may be consistent with this model for initiation of B cell immortalization [30,31].

Our studies show that the first 150 amino acids (aa) of EBNA2 are important for initiation. So, what does the EBV E2 N-terminus do in human B cells that the rhE2 N-terminus cannot? Previous studies have indicated that the first 58 aa have transactivating activity when fused to a DNA binding protein, but deletion mutants of EBNA2 indicate that the first 58 aa are not required for primary B cell immortalization [32,33]. The region identified here encompasses residues 1–150 of EBNA2 which also includes domains that facilitate interaction with Nur77 (aa123-147) and DP103 (aa121-213) [34,35]. These proteins are involved in protecting cells from apoptotic stimuli and regulating RNA metabolism, however neither seems to be required for B cell immortalization [36]. Additionally, the N-terminus of EBNA2 encodes for two self-association, or dimerization, domains (SAD): SAD1, encoded by aa1-58, and SAD2, encoded by aa97-121 [15]. The SADs span 3 CRs and are highly conserved. Friberg *et al*. have recently determined the structure of the N-terminal self-association domain of EBNA2 and found that the residues within the dimerization interface are completely conserved in EBV E2 and rhE2 [37]. However, dimerization is unlikely to be a species-specific event since EBNA2 proteins or mutants produced in bacteria are able to associate, indicating that other (host) proteins are not required for dimerization [15]. Thus, the underlying molecular mechanism for the species-specific effect critical for initiation of B cell immortalization is novel and remains to be defined.

Similarly, the mechanism for the species-specific effect important for maintaining B cell immortalization remains to be uncovered. The region important for maintenance of immortalization (aa295-378) includes CR5 (aa295-307) and CR6 (aa320-326) which facilitate interaction with SKIP and RBP-Jκ, respectively [16,17]. CR5 and CR6 are strongly conserved between EBV E2 and rhE2, but they are not identical (6/10 and 6/7 identical residues). When we constructed E2 mutants where the CR5 or CR6 sequences in EBV E2 were converted to those of rhE2, all mutants could still transactivate Cp to high levels, as well as initiate and maintain human B cell immortalization comparable to wild type EBV E2. Thus, the species-specific effect for maintenance that maps to this region, does not appear to be directly associated with CR5 or CR6. It is also separate from aspartate 442 which is important for EBNA2 mediated survival of ER/EB2-5 cells and growth differences between EBV type I and II EBNA2s [38,39]. The transcription factor PU.1 and the cellular chaperon nucleophosmin 1 (NPM1) both regulate EBNA2 function and bind within the species-specific domain for maintenance (aa310-376 and aa300-360 respectively), but PU.1 and NPM1 proteins from humans and NHPs differ in only one amino acid, making these interactions unlikely candidates for driving the virus adaptation leading to a species-specific effect [40,41]. A poly arginine-glycine (pRG) domain (aa345-356) distal to CR5 and 6 is also located within the region identified as species-specific for maintenance. In EBV E2, this site is methylated by protein arginine methyltransferase 5 (PRMT5) allowing interaction with the survival of motor neurons (SMN) protein [42,43]. However, the biologic importance of this domain is uncertain as a pRG domain is not present in all LCV E2s and may not be essential for B cell immortalization. A large EBNA2 deletion (aa333-425) including pRG is non-transforming, but a smaller deletion including most of pRG (aa333-354) is transformation competent, though slightly impaired compared to wild type EBV E2 [36,44]. It is difficult to know for certain whether a loss-of-function is due to the loss of an essential domain or due to abnormal protein conformation from the physical loss of amino acid residues. In our experiments, the EBV domain from aa295-378 was able to confer a gain of maintenance function to rhE2. The underlying mechanism for the species-specific maintenance function we identify in EBV E2 from aa295-378 appears to be different from the known functions that map to this region.

Our studies of NHP EBNA2s also identified DR as a contributor to high level EBNA2-induced transcriptional activation, but the ability to transcriptionally activate Cp to high levels was not necessary to maintain B cell immortalization and did not correlate with RBP-Jκ binding. It is important to recognize that Cp was used to screen for EBNA2-induced transactivation, but Cp may not necessarily represent all EBNA2-responsive targets. For example, LMP1 promoter activation depends on PU.1, but activation of Cp does not [40]. Thus, there may be Cp-like and non-Cp-like EBNA2-responsive promoters, and our studies do not rule out the possibility that high level transactivation of non-Cp-like promoters, and high level expression of those gene products, is important for maintenance of B cell immortalization. On the other hand, high level transactivation of Cp-like promoters may be required, in combination with the N-terminus, for initiation of immortalization, a model consistent with, but not proven by our current series of chimeras.

A role for DR in EBNA2-induced transactivation has not previously been implicated, and few functions have been attributed to this domain. A phosphorylation site for cdc2/cyclin B1 kinase within the EBV E2 DR (S243) has been described to be phosphorylated during mitosis resulting in decreased EBNA2 transactivation activity [45,46]. This site is conserved in all NHP EBNA2 proteins, but the motif in rhE2 differs from that in EBV E2 (TPPK or SPPR, respectively) raising the possibility that the impaired transactivation of the rhE2 DR in human B cells may be due to excessive phosphorylation of rhE2 versus EBV E2 by cdc2/cyclin B1. We were able to reproduce the phosphorylation of both EBV E2 and rhE2 in human B cells by nocodazole-induced mitotic arrest. However, EBV E2 and rhE2 were phosphorylated to similar proportions, and we found no change in Cp transactivation associated with nocodazole treatment (S2 Fig). Thus, cdc2/cyclin B1-induced phosphorylation cannot explain the species-specific transactivation properties mapped to DR, and the underlying mechanism of how DR contributes to transactivation remains to be elucidated.

Evolution of LCV in OW-NHP and hominoids has provided a natural set of EBNA2 variants that efficiently immortalize B cells from their own species, but are defective for immortalization across species in human B cells. It is likely that other latent infection proteins also function in a species-specific manner since recombinant EBV expressing rhE2 could not immortalize rhesus B cells. Other latent infection proteins, i.e., EBNA3A, –3C, and LMP1 are essential for B cell immortalization and show similar sequence divergence among different LCVs as EBNA2. In addition, previous experiments using rhLCV EBNA3s to replace EBV EBNA3s for human B cell immortalization using second site recombination gave negative results [7].

The natural variants of latent infection proteins represent a unique model system for investigating EBV molecular biology. Understanding the barriers to LCV-induced B cell immortalization across species can provide novel insights into the mechanisms by which LCV manipulate key cell determinants to immortalize B cells, persist for life in the host, and induce B cell malignancies.

## Materials and methods

### Ethics statement

Human PBMC were isolated from peripheral blood obtained from healthy adult volunteers providing informed written consent under a human research protocol approved by the Institutional Review Board of Brigham and Women’s Hospital (protocol #1999P002609/BWH).

### Cell culture and antibodies

The EBV-negative B lymphoma cell lines Louckes and BJAB, the EBV-positive line P3HR-1, and the human epithelial cell line 293T were cultured in RPMI medium supplemented with 10% fetal calf serum (FCS), 100 U/ml penicillin, and 100 μg/ml streptomycin [47–50]. All cell lines were originally obtained from Elliott Kieff. Medium for ER/EB2-5 cells (kindly provided by Bettina Kempkes and Paul Ling) contained an additional 1 μM β-estradiol (Sigma) [12]. For selection of cells containing recombinant viruses 2 μg/ml puromycin was added to the culture medium.

PBMC were isolated from heparinized peripheral blood by density centrifugation over a Ficoll-Hypaque gradient, washed 3 times with phosphate buffered saline (PBS) and cultured in RPMI supplemented with 10% FCS, 100 U/ml penicillin, 100 μg/ml streptomycin, 20 mM HEPES, 4 mM Glutamine, 1 mM Oxaloacetic acid, 1.4 μM insulin, and 500 μM pyruvic acid. Cyclosporin A (0.5 μg/ml) was added for the first week after infection.

### Plasmids and DNA cloning

EBV BAC mutants were created by lambda recombinase mediated homologous recombination. To ensure identical sequences upstream and downstream of every EBNA2 tested, a shuttle-plasmid was constructed that served as donor for the recombination reaction. It included 50 nucleotides (nt) homologous to the sequence upstream of the EBNA2 ORF (-59 to -10 relative to the ATG), two restriction sites (MluI and BsiWI) separated by a 9 nt linker, a chloramphenicol resistance gene flanked by FRT sites derived from pKD3 (CamR-FRT), and 50 nt homologous to the sequence downstream of EBNA2 starting with the stop codon, and was created by PCR amplification of CamR-FRT from pKD3 [51]. NHP EBNA2 genes were PCR amplified, inserted into the MluI/BsiWI sites, and sequenced. An EcoRI fragment containing flanking 50 bp homology region, linkers, EBNA2 genes and CAM-FRT was used for recombination. First, the EcoRI fragment from the parental shuttle plasmid (without EBNA2) was used for recombination with the wild type EBV BAC, MD1, to create MD1ΔE2 (Fig 1A) [51,52]. Then, EBNA2-containing EcoRI shuttle fragments were used for recombination with MD1ΔE2. Two independent BAC recombinants were used to generate each rEBV (one BAC recombinant for rEBV-cyE2). CamR-FRT was subsequently removed by FLP-mediated recombination from all BACs [51]. Integrity of all BACs was confirmed by BamHI restriction digest and Southern Blot. For EBNA2 expression plasmids, the multiple cloning site of pSG5 (Stratagene) was expanded to include MluI and BsiWI sites and EBNA2 genes were transferred from shuttle plasmids to the modified pSG5 plasmid.

EBV/rhLCV EBNA2 chimeras were constructed by small overlap extension PCR. First, EBV-E2 and rhE2 regions were PCR amplified in separate reactions. Inner primers amplifying the site of the fusion contained additional 20 nt complementary to the other EBNA2 product, creating a 40 nt homology. Primary PCR products were purified, combined, and extended in a 10 cycle PCR reaction in which the 40 nt overlap served as primers. The extension reaction was diluted 1:20 and used as the template in a PCR with the outer primers only. The final amplification product was cloned into the shuttle plasmid, sequenced, and used for BAC recombination as described above. For rhE2-mutants containing EBV E2 DR and/or CR5-6, a conserved MfeI-site in EBV E2 (nt 880–885) and rhE2 (nt 1168–1173) was utilized for cloning of fragments between chimeras.

Plasmid pSG-EBV-E2-WW/AA encodes a RBP-Jκ binding mutant of EBV E2, in which tryptophanes 323 and 324 in the RBP-Jκ binding motif of EBNA2 have been substituted with alanines. It was created by site-directed mutagenesis of plasmid pSG-EBV-E2 using the Quik Change II XL site-directed mutagenesis kit (Stratagene/Agilent Technologies) according to manufacturer’s instructions.

### Production of rEBV and infection of human B cells

MD1-EBNA2 BAC DNA was transfected into P3HR-1 cells as a helper cell line. Stable clones were selected with puromycin and confirmed by EBNA2 immunoblot. Virus production was induced by culturing cells in RPMI containing 20 ng/ml phorbol 12-myristate 13-acetate (TPA) and 3 mM butyrate. Virus containing supernatants were collected after 7 days and filtered using a 0.45 μm filter.

Louckes cells were infected with 1ml virus supernatant/5x10^6^ cells and either used for EBNA2 immunoprecipitation after 2 days or plated into 50 microtiter wells at 1x10^5^ cells/well and selected for stable infection with puromycin.

For ER/EB2-5 infection, 10^7^ cells were incubated with 2 ml of virus in the presence of estrogen and plated into 2x 50 microtiter wells. After 24 h the media was changed to i) RPMI/10% FCS only or ii) media supplemented with β-estradiol and puromycin. Plates were incubated for a total of 4–6 weeks, and media was refreshed 2 weeks after infection.

PBMCs were infected with 1 ml rEBV/10^7^ cells in supplemented RPMI with Cyclosporin A (CsA) and plated into microtiter plates at 2x10^5^ cells/well. After 1 week the medium was replaced with supplemented RPMI without CsA. Positive wells were scored after 6 weeks in culture.

Recombinant EBV-E2 or rEBV-baE2 in transformed human LCLs was confirmed by PCR following DNA-isolation using the DNeasy Blood and Tissue kit (Qiagen). Species-specific primers located 100 bp upstream of the EBNA2 stop codon together with an EBV downstream primer (100 bp downstream of the EBNA2 stop codon) were used to detect viral DNA. Primers amplify a 200bp product from endogenous/wild type EBV or a 280 bp product from rEBVs, which contain the scar region.

### EBNA2 immunoprecipitation

Cells were washed with PBS, lysed with 1 ml modified Ripa buffer (50 mM Tris/HCl pH 7.4, 150 mM NaCl, 1 mM EDTA, 1 mM NaF, 1% NP-40, 0.25% Sodium deoxycholate, 1 mM PMSF, 1x protease inhibitor mix [Roche]) on ice for 30 min and insoluble material was removed by centrifugation. Lysates were precleared by incubation with protein G sepharose beads (GE Healthcare) for 15 min on ice and beads were removed by centrifugation. Precleared lysates were incubated with 5–10 μg PE2 antibody overnight at 4°C. The next day samples were incubated with protein G sepharose beads for 2 hours at 4°C. Beads were washed three times with cold PBS, and bound proteins were analyzed by SDS-PAGE and immunoblot.

### SDS-PAGE and immunoblot

Proteins from cell lysates were separated by SDS-PAGE, transferred to nitrocellulose and immunoblotted with the following primary antibodies: 1) Murine monoclonal antibody (mab) PE2 (anti-EBNA2), 2) murine mab S12 (anti-LMP1), 3) murine mab anti-GAPDH (Millipore), 4) murine mab anti-γTubulin antibody (Sigma), or 5) EBV-immune human serum (anti-EBNA1).

### CD23 staining of human LCLs

For surface detection of CD23, cells were washed with PBS and incubated with mouse-anti-CD23 (MHM6) ascites fluid (1:1,000 in PBS/3% BSA) for 45 min on ice. Cells were washed 3 times with PBS/1%FCS and incubated with fluorescein isothiocyanate (FITC)-labeled goat-anti-mouse antibody (Jackson Laboratories) for 45 min on ice. After 3 washes with PBS/1%FCS, cells were analyzed using a FACScalibur cytometer (BD Biosciences).

### B cell transfection and luciferase assays

P3HR-1 cells were split the day before transfection. The next day, 5x10^6^ cells were mixed with 5 μg GFP expression plasmid, 5 μg Renilla control plasmid (a gift from Karl Münger), 10 μg pLucCp ([53], kindly provided by Elliott Kieff), and 30 μg EBNA2 expression plasmid or empty control and adjusted to a total volume of 400 μl with RPMI. For experiments with EBNA-LP, 30 μg EBNA-LP expression plasmid were included. Cells were transferred to 0.4 cm cuvette, electroporated using the Bio-Rad GenePulser II at 200 V and 0.95 μF, and recovered in 10 ml media for 2 days. Then, cells were washed with PBS and 5x10^5^ cells were analyzed for GFP expression by flow cytometry to ensure similar transfection rates, and 1x10^6^ cells were set aside for immunoblot analysis.

For Luciferase assays 5x10^5^ cells were lysed in 100 μl lysis buffer and analyzed using the Dual-Luciferase Reporter Assay System (Promega) according to manufacturer’s instructions. Luciferase signals were first normalized to the corresponding Renilla signal, and then to the empty control to get the fold change over background. Luciferase levels are shown as mean + standard deviation.

### RBP-Jκ binding assay

Myc-tagged RBP-Jκ was expressed in 293T cells. Cells grown in a 15 cm tissue culture dish were transfected with 50 μg pBabe-myc-Jκ (kindly provided by Jon Aster) using 150 μg polyethylenimine (Polysciences Inc.). After 3 days, cells were washed with cold PBS and lysed with 5 ml modified Ripa buffer in the dish. After an incubation of 30 minutes on ice, the lysate was transferred to a test tube, sonicated and insoluble material removed by centrifugation. The lysate was split into 1 ml aliquots and incubated with similar amounts of EBNA2 proteins produced using the TNT Lysate Coupled Transcription/Translation kit (Promega) according to manufacturer’s instructions. Mixed samples were incubated for 3–4 hr at 4°C to allow binding, and then incubated with 2 μg mouse anti-myc antibody (9E10, Thermo Scientific) overnight at 4°C. The next day samples were incubated with protein G sepharose beads for 2 hours at 4°C. Beads were washed 3x 10min with modified Ripa buffer on ice, and bound proteins were analyzed by SDS-PAGE and immunoblot with an HRP-labeled PE2 antibody. Blots were stripped and reprobed with mouse anti-myc antibody and HRP-labeled goat antibody specific for murine immunoglobulin light chain. Based on signal intensities of the precipitates and respective inputs, the percentage of recovered (EBNA2) and precipitated (myc-RBP-Jκ) protein was calculated and amounts of bound EBNA2 were normalized to the amount of myc-RBP-Jκ precipitated. Results are shown as relative EBNA2 binding compared to EBV E2 (100%).

## Supporting information

S1 Fig Nuclear localization of EBNA2 chimeras. 293T cells were transfected with expression plasmids for EBNA2 chimeras and wild type controls. After 2 days cells were fixed with 4% Paraformaldehyde (Electron Microscopy Sciences), permeabilized with 0.1% Triton X, and stained with FITC-labeled PE2 antibody. GFP expressing cells served as control for pan-cellular localization. EBV E2, rhE2, and all EBNA2 chimeras localized exclusively to the nucleus and formed a speckled pattern that is typical for EBV E2.
(TIF)

S2 Fig Effect of mitotic arrest on EBNA2 transactivation activity. P3HR-1 cells were transfected with a Cp-Luciferase reporter, a Renilla control, and EBNA2 expression plasmids or empty control. After 24h half of the cells were incubated with 100ng/ml nocodazole (Sigma) to induce mitotic arrest; the other half was incubated with DMSO as vehicle control. Two days after transfection cells were used for cell cycle analysis, immunoblot, and Luciferase detection. A) For cell cycle analysis, 5x10^5^ cells were washed with PBS and fixed in 70% Ethanol for 1h at 4°C. Samples were washed twice with PBS, treated with RNase A (Qiagen; 10μg/ml for 15min), stained with propidium iodide (100μg/ml), and analyzed by flow cytometry. Nocodazole increased the number of cells in G2/M phase (percentages shown in histograms) from approximately 28% to 78% in each transfection, indicating successful mitotic arrest. B) Nocodazole and control treated cells were analyzed by immunoblot using PE2. Mitotic arrest induced appearance of slower migrating forms of both EBV E2 and rhE2 (asterisks) consistent with phosphorylation. The relative amount of phosphorylated protein is comparable in EBV E2 and rhE2 expressing cells. C) Luciferase expression was measured as described for Fig 4. No difference in EBV E2 or rhE2 induced Cp transactivation was observed between nocodazole treated and control cells (EBV E2 n = 2, rhE2 n = 1).
(TIF)
