# Supplementary figures and images for: Species-specific functions of Epstein-Barr virus nuclear antigen 2 (EBNA2) reveal dual roles for initiation and maintenance of B cell immortalization
Source: PLoS Pathog. 2017 Dec 20;13(12):e1006772. doi: 10.1371/journal.ppat.1006772 (PMC5754137; doi:10.1371/journal.ppat.1006772)

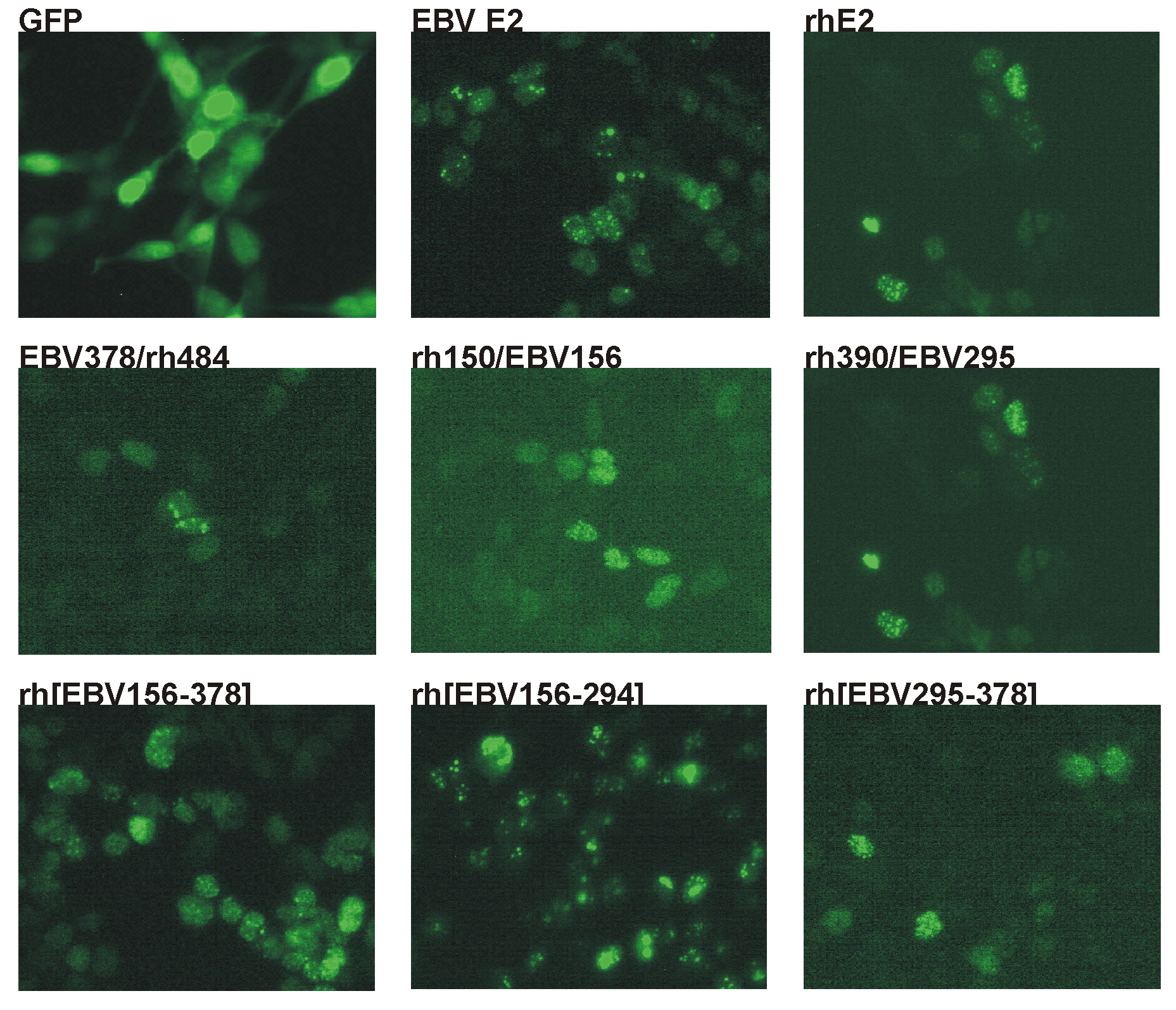

Supplement: S1 Fig — 293T cells were transfected with expression plasmids for EBNA2 chimeras and wild type controls. After 2 days cells were fixed with 4% Paraformaldehyde (Electron Microscopy Sciences), permeabilized with 0.1% Triton X, and stained with FITC-labeled PE2 antibody. GFP expressing cells served as control for pan-cellular localization. EBV E2, rhE2, and all EBNA2 chimeras localized exclusively to the nucleus and formed a speckled pattern that is typical for EBV E2. (TIF) [file ppat.1006772.s001.tif]

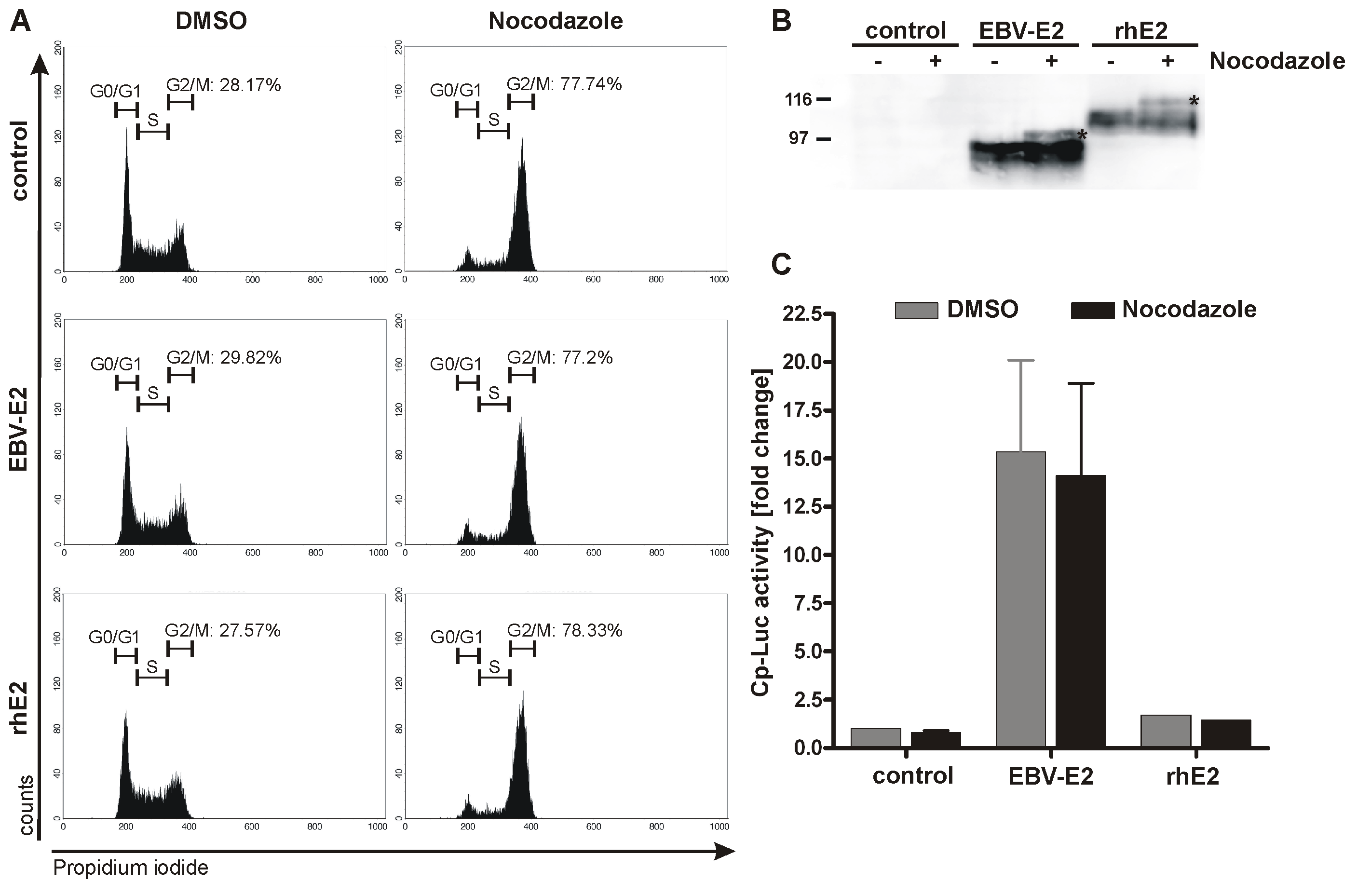

Supplement: S2 Fig — P3HR-1 cells were transfected with a Cp-Luciferase reporter, a Renilla control, and EBNA2 expression plasmids or empty control. After 24h half of the cells were incubated with 100ng/ml nocodazole (Sigma) to induce mitotic arrest; the other half was incubated with DMSO as vehicle control. Two days after transfection cells were used for cell cycle analysis, immunoblot, and Luciferase detection. A) For cell cycle analysis, 5x10^5^ cells were washed with PBS and fixed in 70% Ethanol for 1h at 4°C. Samples were washed twice with PBS, treated with RNase A (Qiagen; 10μg/ml for 15min), stained with propidium iodide (100μg/ml), and analyzed by flow cytometry. Nocodazole increased the number of cells in G2/M phase (percentages shown in histograms) from approximately 28% to 78% in each transfection, indicating successful mitotic arrest. B) Nocodazole and control treated cells were analyzed by immunoblot using PE2. Mitotic arrest induced appearance of slower migrating forms of both EBV E2 and rhE2 (asterisks) consistent with phosphorylation. The relative amount of phosphorylated protein is comparable in EBV E2 and rhE2 expressing cells. C) Luciferase expression was measured as described for Fig 4. No difference in EBV E2 or rhE2 induced Cp transactivation was observed between nocodazole treated and control cells (EBV E2 n = 2, rhE2 n = 1). (TIF) [file ppat.1006772.s002.tif]
